# Supplementary material for: Modularity and predicted functions of the global sponge-microbiome network
Source: Nat Commun. 2019 Mar 1;10:992. doi: 10.1038/s41467-019-08925-4 (PMC6397258; doi:10.1038/s41467-019-08925-4)
Supplement: Supplementary file 1 — Supplementary Information [file 41467_2019_8925_MOESM1_ESM.pdf]

Modularity and predicted functions of the global sponge-microbiome network

Lurgi et al.

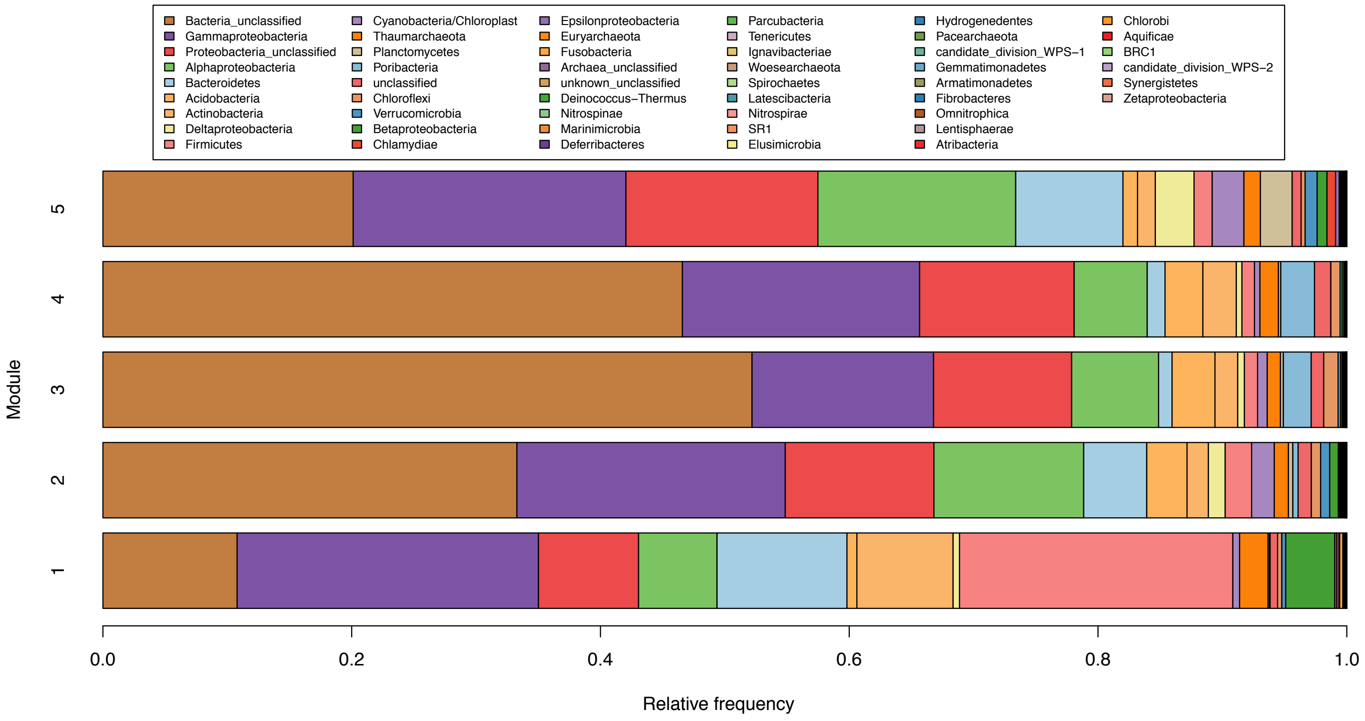

Supplementary Figure 1. Taxonomic profile of microbial communities within the modules of the global sponge-microbiome network. Relative frequencies of individual OTU phyla (as the fraction of OTUs from each phyla) found within the microbiome associated to the modules of the global sponge-microbiome network. Phyla in the legend are presented in the same order as in the boxplot (from left to right). The phylum Proteobacteria is shown as individual classes (including unclassified members of that phylum - Proteobacteria_unclassified).

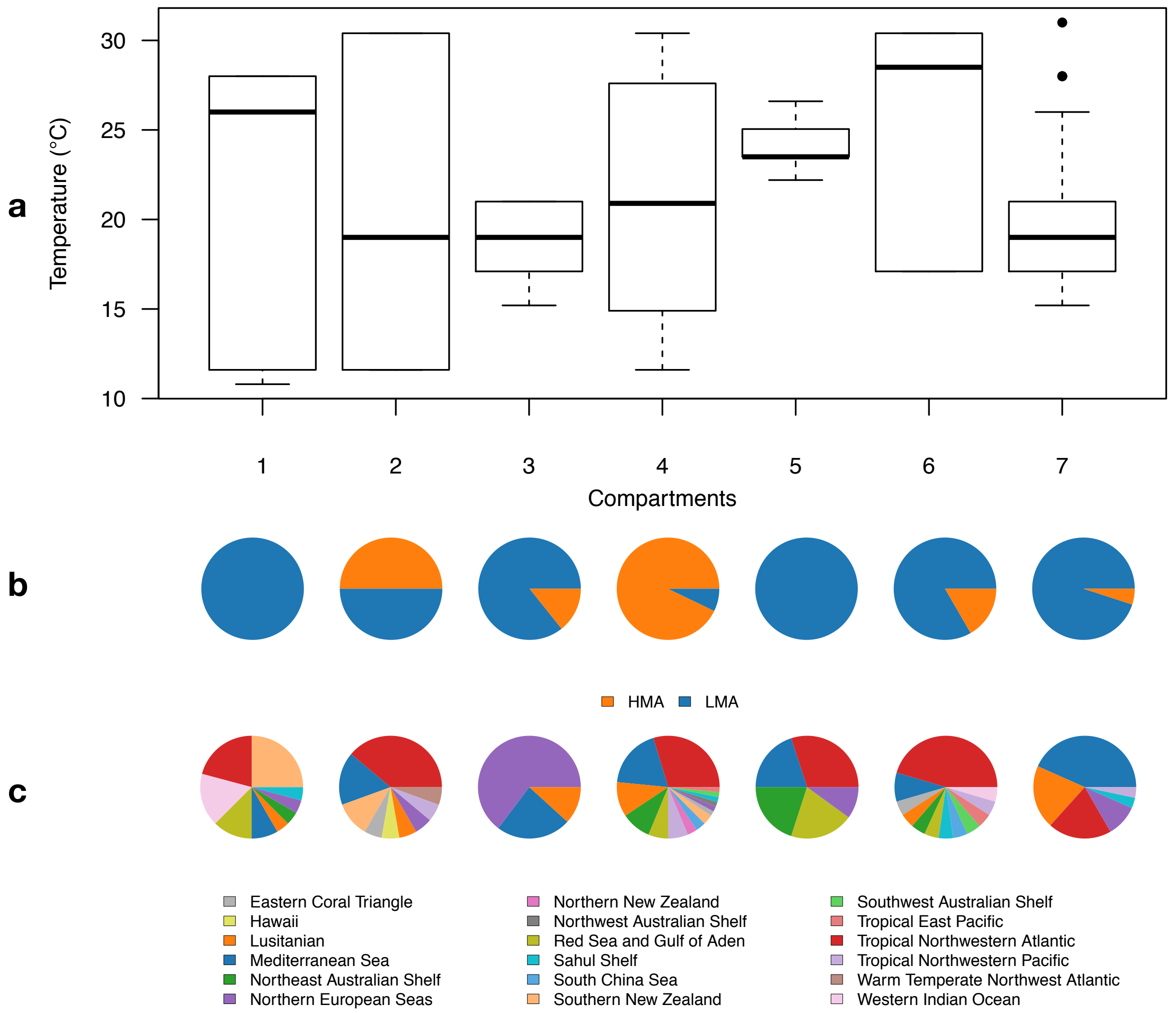

Supplementary Figure 2. Relationship of environmental factors and host type with the modularity of the sponge-core microbiome network. (a) Distribution of temperatures (in degrees Celsius) from sponge sampling sites across network modules identified on the sponge-core microbiome network. Thick line in the middle of boxes represent the median values; box limits are lower and upper quartiles; whiskers, 1.5x interquartile range; points, outliers. (b) Composition of host type (high vs. low microbial abundance sponges) per module. (c) Composition of marine ecoregions from where the samples were collected within each module.

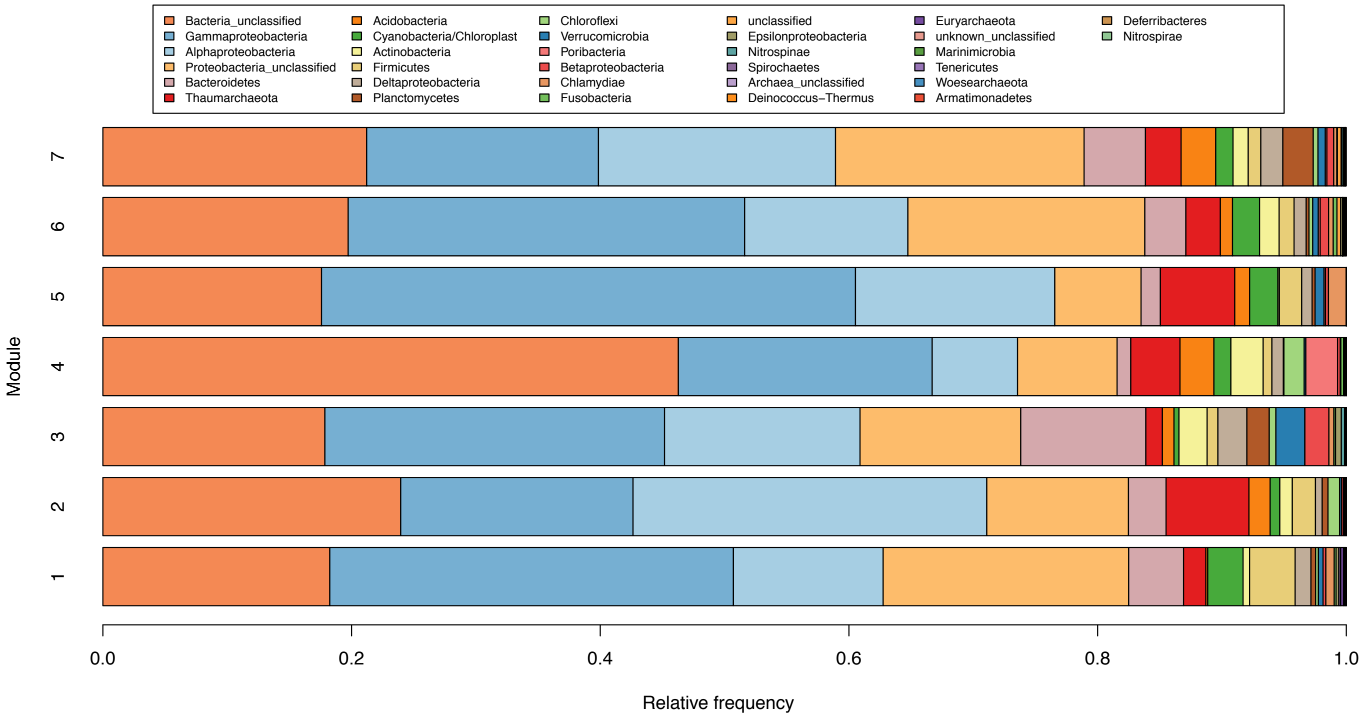

Supplementary Figure 3. Taxonomic profile of microbial communities within the modules of the sponge-core microbiome network. Relative frequencies of individual OTU phyla (as the fraction of OTUs from each phyla) found within the microbiome associated to the modules of the sponge-core microbiome network. Phyla in the legend are presented in the same order as in the boxplot (from left to right). The phylum Proteobacteria is shown as individual classes (including unclassified members of that phylum - Proteobacteria_unclassified).

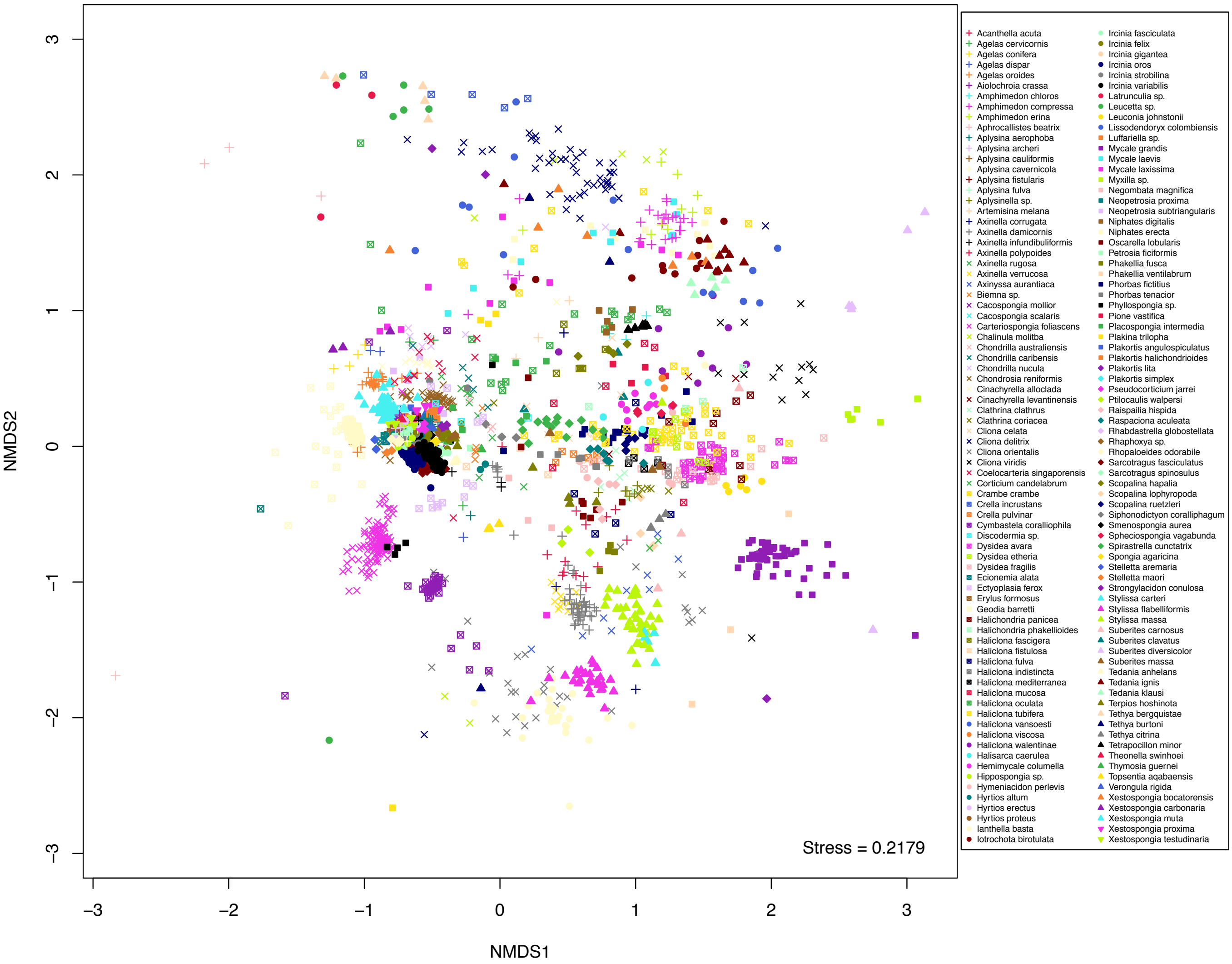

Supplementary Figure 4. Community similarity of microbial communities in sponges. Clustering was performed using non-metric multidimensional scaling (NMDS) over Bray Curtis distances between microbial community samples. Environmental factors were fitted to this ordination and revealed that sponge species identity is the major driver of microbial composition (r^2 = 0.9025, p-value = 0.001), followed by marine ecoregion of the sampling location (r^2 = 0.3141, p-value = 0.001), and to a lesser extent depth (r^2 = 0.0706, p-value = 0.001) and water temperature (r^2 = 0.033, p-value = 0.002). Host type shows a non-significant relationship to this ordination (r^2 = 2e-04, p-value = 0.673).

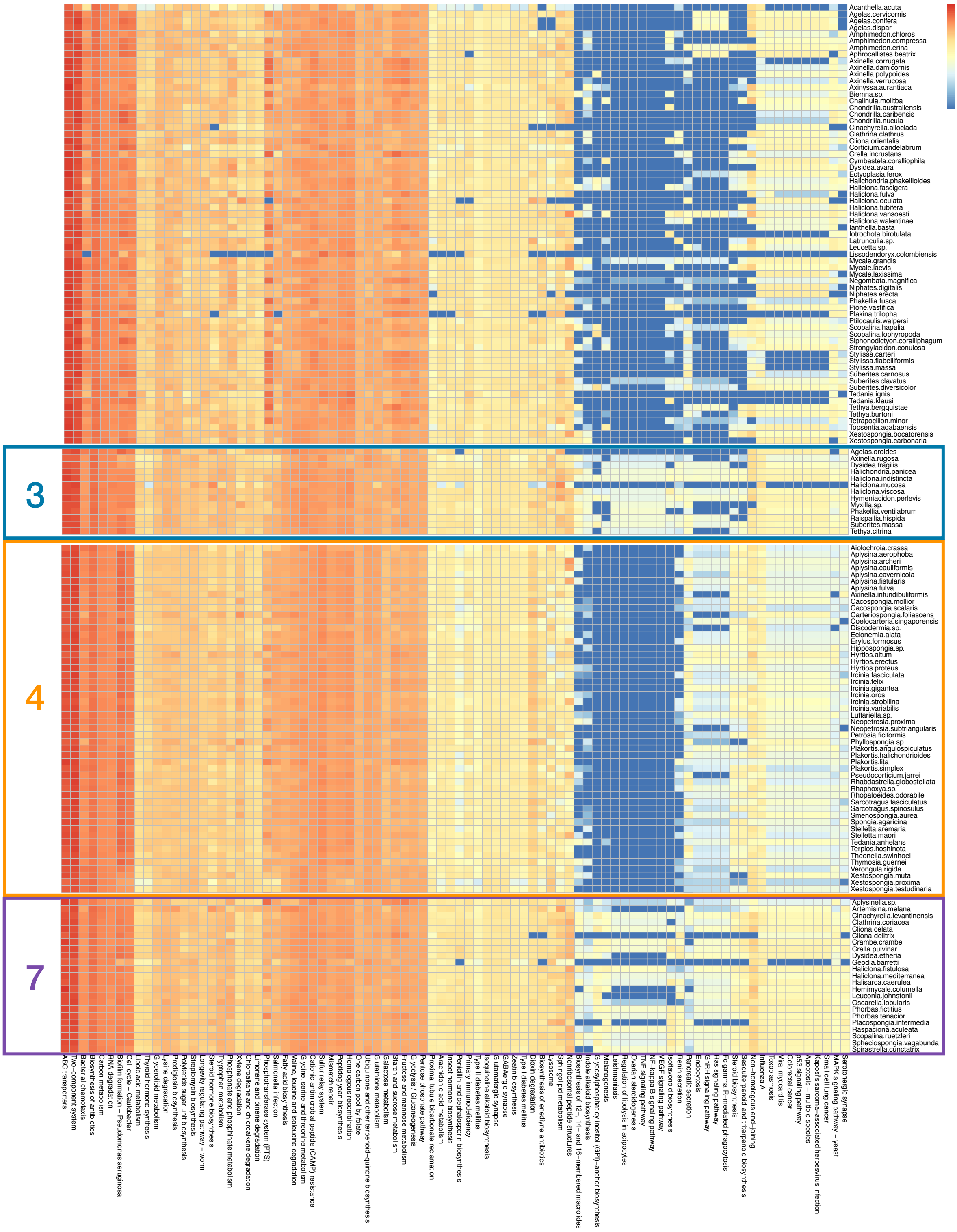

Supplementary Figure 5. Splitting of predicted functions across modules of the global sponge-core microbiome network. Predicted functional profiles of each sponge species considered in this study (rows in heatmap) was obtained by mapping the 16S rRNA gene sequences of core OTUs to their predicted metabolic functions (columns). Colours represent

log2-transformed relative abundances, rescaled by the minimum value, of each function within each sponge host (colours). Function abundances were ultimately derived from the relative abundances of the corresponding OTUs found within the sponges. Predicted functional capabilities of modules 3, 4 and 7 are highlighted. These were identified to be significantly different from each other and the other network modules by indicator species analysis (see Methods). Other network modules are grouped in the remaining section of the heatmap (top).

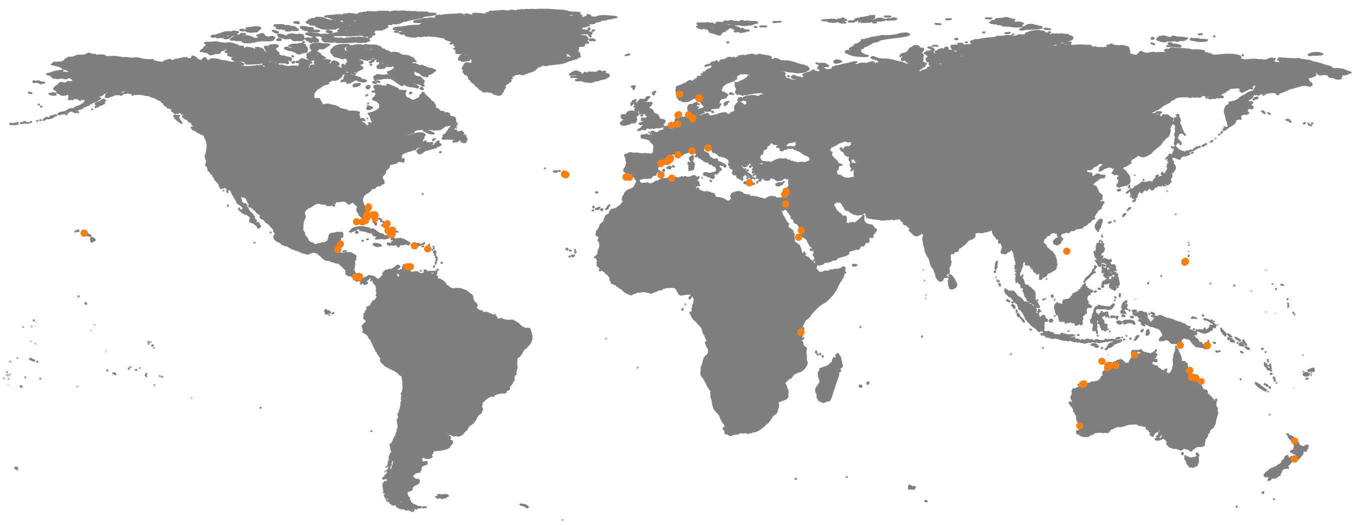

Supplementary Figure 6. Geographical distribution of the sampling locations of marine sponges analysed in this study. 156 sponge species were sampled across 202 locations across the globe (orange points in the map) to characterise their associated microbial communities as part of the Sponge Microbiome Project (SMP). This map was generated using the ggplot2 graphics library in R.

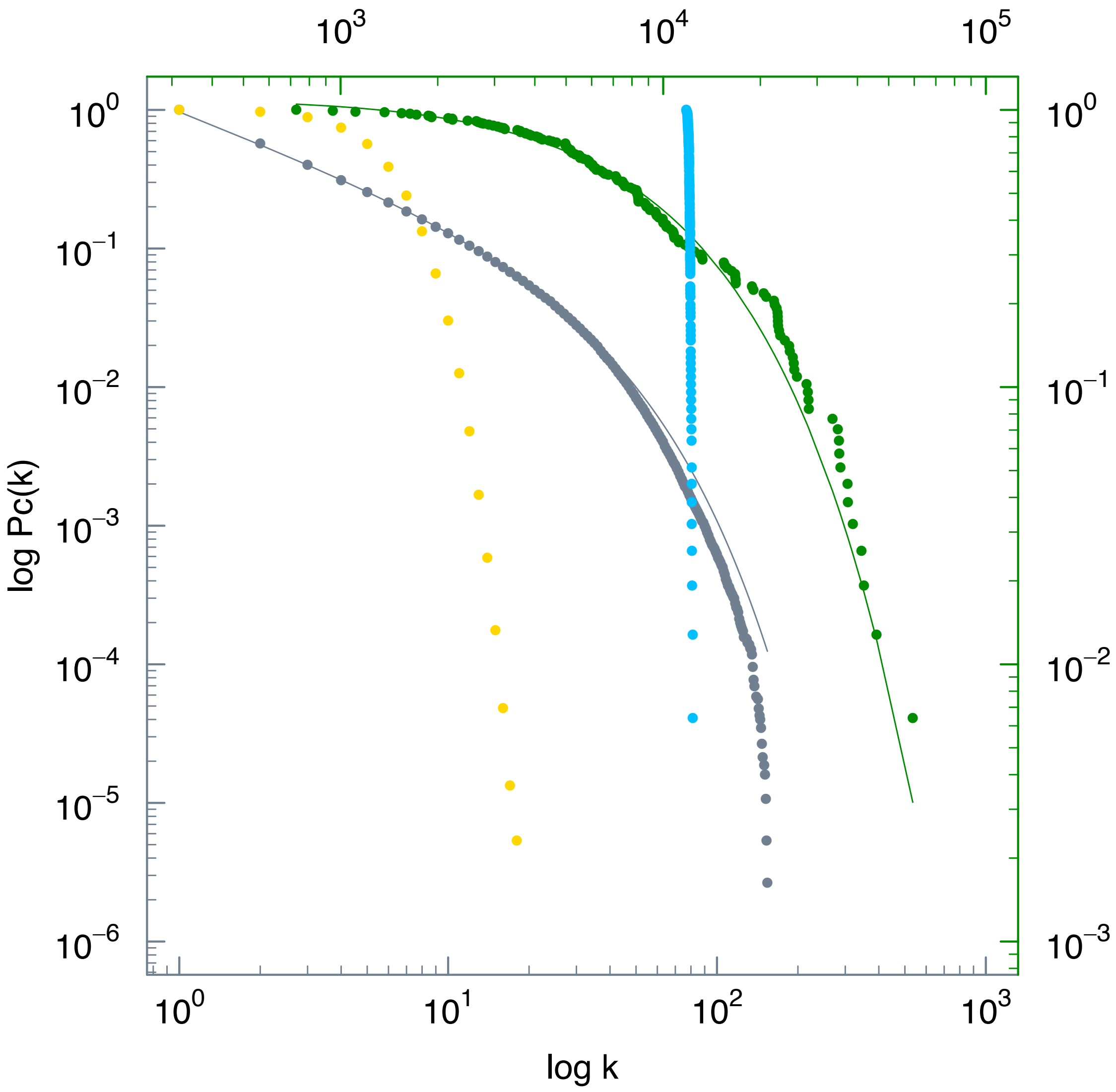

Supplementary Figure 7. Cumulative degree distributions for OTUs (grey dots, bottom and left axes) and sponges (green dots, top and right axes). Grey dots (and axis) correspond to the number of different host species (k) that contain a given OTU, represented as the cumulative probability of finding an OTU in the network with k or less-associated hosts (Pc(k)). Green dots (and axis) correspond to the number of different OTUs (k) found in a given host species, represented as the cumulative probability of finding a sponge host with k or less-associated OTUs (Pc(k)). Yellow and blue dots correspond to degree distributions from a random network in which the number of nodes and links from the empirical network are kept constant, for OTUs and sponges, respectively.

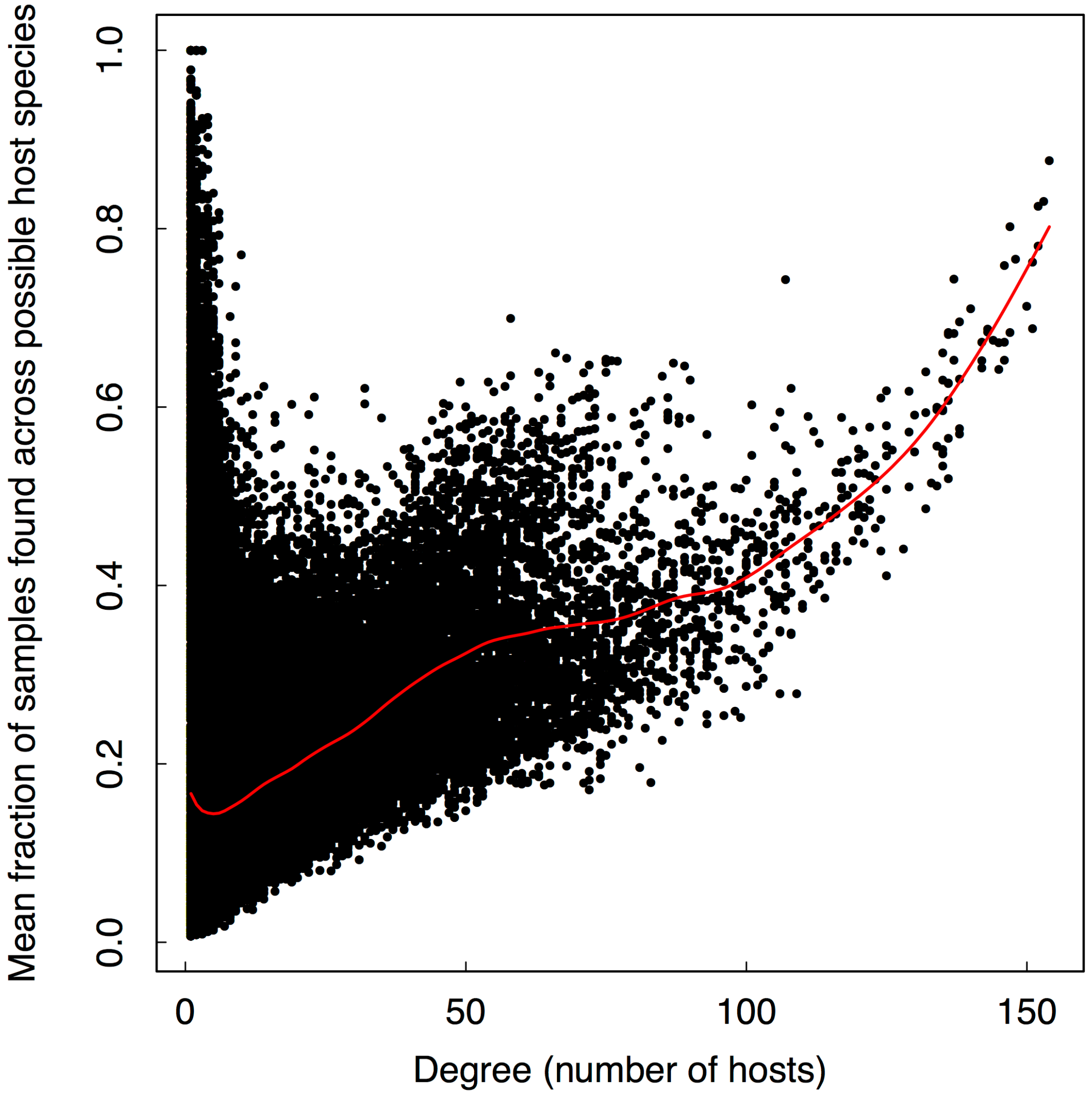

Supplementary Figure 8. Prevalence of OTUs across different degrees of host-specificity. Number of host species (degree) containing a given bacterial OTU in the global sponge-microbiome network plotted against the fraction of individual samples where each OTU has been found among all the samples from their known host species. Each point represents an OTU and the line is a smoothing spline fit to the data.

Supplementary Table 1. Seawater temperature as a driver of modular organisation of the global sponge-microbiome network. Results of pairwise comparisons, using Nemenyi test with chi-squared approximation for independent samples, between the distributions of temperature within each of the 5 modules identified from the global network. Magnitude of the statistic quantifies the strength of the relationship, with larger values implying larger differences between groups. As shown by the p-values, all groups (i.e., distributions of temperatures of sampling locations) are different to each other except for the pair of groups comprised of modules 2 and 3.

Pairs	*Statistic*	*p-value*
1 = 2	122.000561	< 2e-16
1 = 3	160.425503	< 2e-16
1 = 4	22.685219	0.00015
1 = 5	72.962853	5.30e-15
2 = 3	5.906785	0.20622
2 = 4	143.887945	< 2e-16
2 = 5	21.636896	0.00024
3 = 4	223.907922	< 2e-16
3 = 5	53.669641	6.20e-11
4 = 5	62.172636	1.00e-12

Supplementary Table 2. Depth at which the host (sponge) is found does not show a relationship with the modularity of the global sponge-microbiome network. Results of pairwise comparisons, using Nemenyi test with chi-squared approximation for independent samples, between the distributions of sampling depths within each of the 5 modules identified in the global network. Magnitude of the statistic quantifies the strength of the relationship, with larger values implying larger differences between groups. As shown by the p-values, no significant differences occur between groups except for module 1, for which almost all sponges come from a single sampling location (see main manuscript for a discussion on the possible causes of this).

Pairs	***Statistic***	***p-value***
1 = 2	28.660749	9.20e-06
1 = 3	32.016104	1.90e-06
1 = 4	25.890177	3.30e-05
1 = 5	24.403018	6.60e-05
2 = 3	0.2343319	0.99
2 = 4	0.5630954	0.97
2 = 5	2.2726117	0.69
3 = 4	1.8208615	0.77
3 = 5	5.4239606	0.25
4 = 5	0.5171221	0.97

Supplementary Table 3. Microbial phylogenetic signal in the modularity of the global sponge-microbiome network. Unweighted UniFrac analyses used to assess the significance of the phylogenetic fingerprint of the sponge-associated microbial communities in the modular structure detected in the global network. All comparisons were statistically significant.

Pairs	*UWScore*	*p-value (UWSig)*
1-2	0.893475	<0.001
1-3	0.929143	<0.001
2-3	0.800973	<0.001
1-4	0.927007	<0.001
2-4	0.804092	<0.001
3-4	0.766469	<0.001
1-5	0.946554	<0.001
2-5	0.853357	<0.001
3-5	0.880384	<0.001
4-5	0.896039	<0.001

Supplementary Table 4. Module membership and sponge type for sponges in the sponge-core microbiome network. Sponge type has been classified as LMA, HMA, or NA for low-microbial abundance, high-microbial abundance, and not unclassified species respectively. Module 4 is primarily comprised of HMA sponges, while LMA sponges are scattered across the remaining modules.

Sponge species	***Sponge type***	***Module***
Acanthella acuta	LMA	1
Amphimedon chloros	LMA	1
Amphimedon compressa	LMA	1
Amphimedon erina	LMA	1
Cliona orientalis	NA	1
Halichondria phakellioides	NA	1
Haliclona fascigera	LMA	1
Haliclona fulva	LMA	1
Haliclona oculata	LMA	1
Haliclona walentinae	LMA	1
Latrunculia sp.	LMA	1
Leucetta sp.	LMA	1
Lissodendoryx colombiensis	LMA	1
Pione vastifica	LMA	1
Plakina trilopha	NA	1
Ptilocaulis walpersi	LMA	1
Scopalina hapalia	NA	1
Scopalina lophyropoda	LMA	1
Strongylacidon conulosa	NA	1
Suberites clavatus	LMA	1
Tethya burtoni	NA	1
Tetrapocillon minor	LMA	1
Agelas cervicornis	HMA	2
Agelas conifera	HMA	2
Agelas dispar	HMA	2
Aphrocallistes beatrix	LMA	2
Axinyssa aurantiaca	NA	2
Chondrilla caribensis	HMA	2

Chondrilla nucula	NA	2
Cinachyrella alloclada	HMA	2
Clathrina clathrus	LMA	2
Corticium candelabrum	HMA	2
Crella incrustans	LMA	2
Mycale grandis	LMA	2
Mycale laxissima	LMA	2
Suberites carnosus	LMA	2
Tethya bergquistae	NA	2
Xestospongia carbonaria	NA	2
Agelas oroides	HMA	3
Axinella rugosa	LMA	3
Chondrosia reniformis	HMA	3
Dysidea fragilis	LMA	3
Halichondria panicea	LMA	3
Haliclona indistincta	LMA	3
Haliclona mucosa	LMA	3
Haliclona viscosa	LMA	3
Hymeniacidon perlevis	LMA	3
Myxilla sp.	LMA	3
Phakellia ventilabrum	LMA	3
Raispailia hispida	LMA	3
Suberites massa	LMA	3
Tethya citrina	LMA	3
Aiolochroia crassa	HMA	4
Aplysina aerophoba	HMA	4
Aplysina archeri	HMA	4
Aplysina cauliformis	HMA	4
Aplysina cavernicola	HMA	4
Aplysina fistularis	HMA	4
Aplysina fulva	HMA	4
Axinella infundibuliformis	LMA	4
Cacospongia mollior	HMA	4
Cacospongia scalaris	HMA	4
Carteriospongia foliascens	LMA	4
Coelocarteria singaporensis	NA	4
Discodermia sp.	NA	4
Ecionemia alata	NA	4

Erylus formosus	HMA	4
Hippospongia sp.	HMA	4
Hyrtios altum	HMA	4
Hyrtios erectus	HMA	4
Hyrtios proteus	HMA	4
Ircinia fasciculata	NA	4
Ircinia felix	HMA	4
Ircinia gigantea	NA	4
Ircinia oros	HMA	4
Ircinia strobilina	HMA	4
Ircinia variabilis	HMA	4
Luffariella sp.	HMA	4
Neopetrosia proxima	HMA	4
Neopetrosia subtriangularis	HMA	4
Petrosia ficiformis	HMA	4
Phyllospongia sp.	LMA	4
Plakortis angulospiculatus	HMA	4
Plakortis halichondrioides	HMA	4
Plakortis lita	NA	4
Plakortis simplex	HMA	4
Pseudocorticium jarrei	HMA	4
Rhabdastrella globostellata	HMA	4
Rhaphoxya sp.	HMA	4
Rhopaloeides odorabile	HMA	4
Sarcotragus fasciculatus	HMA	4
Sarcotragus spinosulus	HMA	4
Smenospongia aurea	HMA	4
Spongia agaricina	HMA	4
Stelletta aremaria	NA	4
Stelletta maori	HMA	4
Tedania anhelans	NA	4
Terpios hoshinota	NA	4
Theonella swinhoei	HMA	4
Thymosia guernei	NA	4
Verongula rigida	HMA	4
Xestospongia muta	HMA	4
Xestospongia proxima	HMA	4
Xestospongia testudinaria	HMA	4

Axinella damicornis	LMA	5
Axinella verrucosa	LMA	5
Cymbastela coralliophila	NA	5
Ianthella basta	LMA	5
Negombata magnifica	LMA	5
Niphates digitalis	LMA	5
Niphates erecta	LMA	5
Siphonodictyon coralliphagum	NA	5
Topsentia aqabaensis	NA	5
Axinella corrugata	LMA	6
Axinella polypoides	LMA	6
Biemna sp.	HMA	6
Chalinula molitba	LMA	6
Chondrilla australiensis	HMA	6
Dysidea avara	LMA	6
Ectyoplasia ferox	HMA	6
Haliclona tubifera	LMA	6
Haliclona vansoesti	LMA	6
Iotrochota birotulata	LMA	6
Mycale laevis	LMA	6
Phakellia fusca	LMA	6
Stylissa carteri	LMA	6
Stylissa flabelliformis	NA	6
Stylissa massa	LMA	6
Suberites diversicolor	LMA	6
Tedania ignis	LMA	6
Tedania klausi	LMA	6
Xestospongia bocatorensis	LMA	6
Aplysinella sp.	LMA	7
Artemisina melana	LMA	7
Cinachyrella levantinensis	LMA	7
Clathrina coriacea	LMA	7
Cliona celata	LMA	7
Cliona delitrix	LMA	7
Cliona viridis	LMA	7
Crambe crambe	LMA	7
Crella pulvinar	NA	7
Dysidea etheria	LMA	7

Geodia barretti	HMA	7
Haliclona fistulosa	NA	7
Haliclona mediterranea	LMA	7
Halisarca caerulea	LMA	7
Hemimycale columella	LMA	7
Leuconia johnstonii	NA	7
Oscarella lobularis	LMA	7
Phorbas fictitius	LMA	7
Phorbas tenacior	LMA	7
Placospongia intermedia	NA	7
Raspaciona aculeata	LMA	7
Scopalina ruetzleri	LMA	7
Spheciospongia vagabunda	LMA	7
Spirastrella cunctatrix	LMA	7

Supplementary Table 5. Heterogeneity in microbial community composition across modules of the sponge core microbiome network. Results of pairwise permutational analysis of variance (PERMANOVA) performed over the OTU table of relative abundances of the sponge core-microbiome network and considering network module as the independent factor for variance comparison. Significance codes: ‘*’ 0.01 ‘.’ 0.05 ‘ ’ 0.1

Pairs	*F statistic*	R^2	*p-value*	*adjusted p-value*	*significance*
1 vs 2	1.792036	0.04741835	0.002	0.007655253	*
1 vs 3	2.909253	0.07882177	0.001	0.004503090	*
1 vs 4	14.992475	0.17234221	0.001	0.004503090	*
1 vs 7	3.159964	0.06700522	0.001	0.004503090	*
1 vs 6	1.846244	0.04519985	0.002	0.007655253	*
1 vs 5	1.89126	0.06122315	0.002	0.007655253	*
2 vs 3	2.575383	0.08423062	0.001	0.004503090	*
2 vs 4	9.610809	0.12710893	0.001	0.004503090	*
2 vs 7	3.57221	0.08592783	0.001	0.004503090	*
2 vs 6	1.961832	0.05611355	0.001	0.004503090	*
2 vs 5	1.880112	0.07556685	0.001	0.004503090	*
3 vs 4	12.922287	0.16799145	0.001	0.004503090	*
3 vs 7	2.821246	0.07267273	0.001	0.004503090	*
3 vs 6	3.34297	0.09734074	0.001	0.004503090	*
3 vs 5	2.830382	0.11877201	0.001	0.004503090	*
4 vs 7	17.821639	0.19408975	0.001	0.004503090	*
4 vs 6	14.120442	0.16987930	0.001	0.004503090	*
4 vs 5	8.614467	0.12740568	0.001	0.004503090	*
7 vs 6	4.204621	0.09301308	0.001	0.004503090	*
7 vs 5	3.350515	0.09753901	0.001	0.004503090	*
6 vs 5	1.975193	0.07060515	0.004	0.014581435	.

Supplementary Table 6. PERMANOVA results for the differences in microbiome composition between modules of the global sponge-microbiome network over rarefied datasets. Reported are the average values and standard deviation across 100 independent rarefaction instances of the raw data (i.e., the OTU table) to 10^4 reads per sample (See *Data rarefaction* in the *Methods* section for details on the rarefaction procedure). All comparisons are significant.

Pairs	*F statistic*	R^2	*p-value*	*adjusted p-value*	*significance*
1 vs 2	2.17 ± 0.005	0.126 ± 0.0002	0.001 ± 0.0003	0.003 ± 0.0009	*
1 vs 3	5.02 ± 0.009	0.088 ± 0.0001	0.001 ± 0	0.003 ± 0.0002	*
1 vs 4	5.11 ± 0.01	0.203 ± 0.0003	0.001 ± 0	0.003 ± 0.0002	*
1 vs 5	2.43 ± 0.006	0.028 ± 0.00007	0.001 ± 0	0.003 ± 0.0002	*
2 vs 3	2.65 ± 0.006	0.048 ± 0.0001	0.001 ± 0.0003	0.003 ± 0.0008	*
2 vs 4	2.96 ± 0.005	0.124 ± 0.0002	0.001 ± 0	0.003 ± 0.0002	*
2 vs 5	1.44 ± 0.002	0.016 ± 0.00002	0.001 ± 0.0006	0.003 ± 0.0017	*
3 vs 4	4.37 ± 0.01	0.07 ± 0.0001	0.001 ± 0	0.003 ± 0.0002	*
3 vs 5	9.78 ± 0.012	0.074 ± 0.00008	0.001 ± 0	0.003 ± 0.0002	*
4 vs 5	5.49 ± 0.006	0.057 ± 0.00005	0.001 ± 0	0.003 ± 0.0002	*
