# Supplementary material for: Modularity and predicted functions of the global sponge-microbiome network
Source: Nat Commun. 2019 Mar 1;10:992. doi: 10.1038/s41467-019-08925-4 (PMC6397258; doi:10.1038/s41467-019-08925-4)
Supplement: Supplementary file 3 — Description of Additional Supplementary Files [file 41467_2019_8925_MOESM3_ESM.pdf]

Description of Additional Supplementary Files

File Name: Supplementary Data 1

Description: Data for reproducing Figure 1 and Supplementary Figure 4 in the manuscript. Subset of the metadata table of the Sponge Microbiome Project (see Data Availability) concerning the samples considered in this study. The table includes water temperature (in degree Celsius), depth (in metres), host type (HMA or LMA), marine ecoregion where the sampling was conducted, and module membership of the sponge species to which each sample belongs to in both, the global and the core microbiome network. This was the data used to generate Figure 1 and Supplementary Figure 4 in the manuscript.
