# Supplementary material for: Modularity and predicted functions of the global sponge-microbiome network
Source: Nat Commun. 2019 Mar 1;10:992. doi: 10.1038/s41467-019-08925-4 (PMC6397258; doi:10.1038/s41467-019-08925-4)
Supplement: Supplementary file 5 — Reporting Summary [file 41467_2019_8925_MOESM5_ESM.pdf]

nature research

Corresponding author(s): Miguel Lurgi (miguel.lurgi@sete.cnrs.fr)

Last updated by author(s): 2019/01/30

Reporting Summary

Nature Research wishes to improve the reproducibility of the work that we publish. This form provides structure for consistency and transparency in reporting. For further information on Nature Research policies, see Authors & Referees and the Editorial Policy Checklist.

Statistics

For all statistical analyses, confirm that the following items are present in the figure legend, table legend, main text, or Methods section.

n/a	Confirmed	
☒	☐	The exact sample size (n) for each experimental group/condition, given as a discrete number and unit of measurement
☒	☐	A statement on whether measurements were taken from distinct samples or whether the same sample was measured repeatedly
☐	☒	The statistical test(s) used AND whether they are one- or two-sided<br>*Only common tests should be described solely by name; describe more complex techniques in the Methods section.*
☐	☒	A description of all covariates tested
☐	☒	A description of any assumptions or corrections, such as tests of normality and adjustment for multiple comparisons
☐	☒	A full description of the statistical parameters including central tendency (e.g. means) or other basic estimates (e.g. regression coefficient) AND variation (e.g. standard deviation) or associated estimates of uncertainty (e.g. confidence intervals)
☐	☒	For null hypothesis testing, the test statistic (e.g. F, t, r) with confidence intervals, effect sizes, degrees of freedom and P value noted<br>*Give P values as exact values whenever suitable.*
☒	☐	For Bayesian analysis, information on the choice of priors and Markov chain Monte Carlo settings
☒	☐	For hierarchical and complex designs, identification of the appropriate level for tests and full reporting of outcomes
☒	☐	Estimates of effect sizes (e.g. Cohen's d, Pearson's r), indicating how they were calculated

Our web collection on statistics for biologists contains articles on many of the points above.

Software and code

Policy information about availability of computer code

Data collection	The Sponge Microbiome Data used in this study was downloaded from are available from the GigaScience repository, GigaDB (DOI:10.5524/100332)<br>Sponge phylogenetic information was downloaded from the Open Tree of Life using the rotl package in R (version 3.0.4)
Data analysis	All statistical analyses performed for this manuscript were done in R version 3.4.4. The appropriate libraries used for each analysis are properly cited in the Methods section of the manuscript. Phylogenetic trees were built with MEGA 7.0.26 and Fasttree 2.1.10. UniFrac distances were calculated using mothur version 1.39.5. Network modularity for bipartite graphs was calculated using the LPAwb+ algorithm available within the bipartite R package version 2.08.

For manuscripts utilizing custom algorithms or software that are central to the research but not yet described in published literature, software must be made available to editors/reviewers. We strongly encourage code deposition in a community repository (e.g. GitHub). See the Nature Research guidelines for submitting code & software for further information.

Data

Policy information about availability of data

All manuscripts must include a data availability statement. This statement should provide the following information, where applicable:

- Accession codes, unique identifiers, or web links for publicly available datasets
- A list of figures that have associated raw data
- A description of any restrictions on data availability

The datasets analysed during the current study have been made available previously in31. Raw sequence data have been deposited in the European Nucleotide Archive (BioProject: PRJEB18736; Accession number: ERP020690). Quality-filtered, demultiplexed fastq files, and QIIME resulting OTU tables are available at the Qiita database: http://qiita.microbio.me (Study ID: 10793). Additional information derived from the sequence data including an OTU abundance matrix, an OTU taxonomic classification table, an OTU representative sequence FASTA file, and the metadata for this dataset, which are the only three data sources used in this study, are available from the GigaScience repository, GigaDB (DOI:10.5524/100332)31. The authors declare that all other data supporting the findings of this study

are available within the article and its Supplementary Information files.

Field-specific reporting

Please select the one below that is the best fit for your research. If you are not sure, read the appropriate sections before making your selection.

☐ Life sciences ☐ Behavioural & social sciences ☒ Ecological, evolutionary & environmental sciences

For a reference copy of the document with all sections, see nature.com/documents/nr-reporting-summary-flat.pdf

Ecological, evolutionary & environmental sciences study design

All studies must disclose on these points even when the disclosure is negative.

Study description	In this study we analysed the dataset available from the sponge microbiome project to investigate the organisation of the sponge microbiome into network modules. The relationship of this organisation to the metabolic functioning of the microbiome was investigated. Additionally, we investigated the potential evolutionary and ecological mechanisms behind the modular organisation of the sponge microbiome by looking at the relationship of three environmental variables (temperature, depth, and membership within the marine ecoregions of the world), the type of host (i.e., whether it is a high or a low microbial abundance sponge), and the phylogenetic structure of both, the microbial communities and the sponge hosts; to the modular organisation of the sponge-microbiome network.
Research sample	This study was based on an existing dataset. This dataset comprises 16S rRNA gene sequencing data from the Sponge Microbiome Project (SMP) dataset, which includes marine sponge samples from many geographical locations across the globe. Raw sequence data was then processed using standard bioinformatic pipelines to produce a table of OTUs presence and abundance across several sponge samples and the associated FASTA files containing these sequences. These data are available from the GigaScience repository, GigaDB (DOI:10.5524/100332), and were the ones used in this study. We used this dataset because we deemed it appropriate to study global patterns of sponge-associated microbial communities, given its size and the geographical scale covered.
Sampling strategy	*Note the sampling procedure. Describe the statistical methods that were used to predetermine sample size OR if no sample-size calculation was performed, describe how sample sizes were chosen and provide a rationale for why these sample sizes are sufficient.*
Data collection	*Describe the data collection procedure, including who recorded the data and how.*
Timing and spatial scale	Spatial scale: global.
Data exclusions	*If no data were excluded from the analyses, state so OR if data were excluded, describe the exclusions and the rationale behind them, indicating whether exclusion criteria were pre-established.*
Reproducibility	*Describe the measures taken to verify the reproducibility of experimental findings. For each experiment, note whether any attempts to repeat the experiment failed OR state that all attempts to repeat the experiment were successful.*
Randomization	*Describe how samples/organisms/participants were allocated into groups. If allocation was not random, describe how covariates were controlled. If this is not relevant to your study, explain why.*
Blinding	*Describe the extent of blinding used during data acquisition and analysis. If blinding was not possible, describe why OR explain why blinding was not relevant to your study.*

Did the study involve field work? ☐ Yes ☒ No

Reporting for specific materials, systems and methods

We require information from authors about some types of materials, experimental systems and methods used in many studies. Here, indicate whether each material, system or method listed is relevant to your study. If you are not sure if a list item applies to your research, read the appropriate section before selecting a response.

Materials & experimental systems

n/a	Involved in the study	
☒	☐	Antibodies
☒	☐	Eukaryotic cell lines
☒	☐	Palaeontology
☒	☐	Animals and other organisms
☒	☐	Human research participants
☒	☐	Clinical data

Methods

n/a	Involved in the study	
☒	☐	ChIP-seq
☒	☐	Flow cytometry
☒	☐	MRI-based neuroimaging
